# *In Vitro* Antibacterial Activity of Essential Oils against *Streptococcus pyogenes*

**DOI:** 10.1155/2013/269161

**Published:** 2013-04-11

**Authors:** Julien Sfeir, Corinne Lefrançois, Dominique Baudoux, Séverine Derbré, Patricia Licznar

**Affiliations:** ^1^PRES LUNAM, Université d'Angers, Laboratoire de Bactériologie-Virologie, UFR Sciences Pharmaceutiques et Ingénierie de la Santé, 16 boulevard Daviers, 49045 Angers, Cedex 01, France; ^2^PRES LUNAM, Université d'Angers, SFR ICAT 4208, EA 3142 GEIHP, 16 boulevard Daviers, 49045 Angers, Cedex 01, France; ^3^S.A. PRANAROM International, 37 Avenue des Artisans, 7822 Ghislenghien, Belgium; ^4^PRES LUNAM, Université d'Angers, SFR QUASAV 4207, EA 921 SONAS, UFR Sciences Pharmaceutiques et Ingénierie de la Santé, 16 boulevard Daviers, 49045 Angers, Cedex 01, France

## Abstract

*Streptococcus pyogenes* plays an important role in the pathogenesis of tonsillitis. The present study was conducted to evaluate the *in vitro* antibacterial activities of 18 essential oils chemotypes from aromatic medicinal plants against *S. pyogenes*. Antibacterial activity of essential oils was investigated using disc diffusion method. Minimum Inhibitory Concentration of essential oils showing an important antibacterial activity was measured using broth dilution method. Out of 18 essential oils tested, 14 showed antibacterial activity against *S. pyogenes*. Among them *Cinnamomum verum, Cymbopogon citratus, Thymus vulgaris CT thymol, Origanum compactum*, and *Satureja montana* essential oils exhibited significant antibacterial activity. The *in vitro* results reported here suggest that, for patients suffering from bacterial throat infections, if aromatherapy is used, these essential oils, considered as potential antimicrobial agents, should be preferred.

## 1. Introduction

Since ten years, the optimization of the use of antibiotics concerns national health agencies that try, through many advertising campaigns and famous slogans, to inform and sensitize people. For example, most cases of tonsillitis are viral and do not need antibiotic treatment. For instance, in about 37% of tonsillitis occurring among children the etiology is bacterial, with *Streptococcus pyogenes* being the most common bacterial etiology [1]. In this particular context, inflamed tonsils have to be treated by antibiotics. Penicillin is the first choice of antibiotic for the treatment of *S. pyogenes *tonsillitis. No *S. pyogenes* resistant to penicillin has been reported. Unfortunately, failures of penicillin treatments to eradicate *S. pyogenes* from tonsillitis/pharyngitis have been reported [2–4].

Among the alternative therapeutic arsenal, the essential oils (EOs) could be an interesting choice against this pathogen; the EOs antiseptic properties have been demonstrated, at least* in vitro* (more than 2000 publications about antimicrobial activity of EOs referenced in PubMed since 2002). In spite of all the information available on EOs, we wanted to evaluate their place in alternative or complementary treatments of *S. pyogenes *tonsillitis.

We carried out *in vitro *experiments to evaluate antibacterial activity of EOs described as active against *S. pyogenes*. Disc diffusion method was performed to test antibacterial activity of 18 EOs; MIC (Minimum Inhibitory Concentration) and MBC (Minimum Bactericidal Concentration) of the 5 most effective EOs were determined. Similar experiments were carried out with amoxicillin, the benchmark treatment in this pathology. 

## 2. Material and Methods

### 2.1. Essential Oils

Eighteen essential oils (*Cinnamomum verum*, *Cymbopogon citratus*, *Origanum compactum*, *Thymus vulgaris* CT thymol, *Satureja montana*, *Eugenia caryophyllus*, *Cymbopogon martinii var*. *motia*, *Cinnamomum camphora* CT linalool, *Mentha piperita*, *Thymus vulgaris* CT thujanol, *Origanum marjorana*, *Lavandula stoechas*, *Melaleuca cajuputi*, *Melaleuca alternifolia*, *Ocimum basilicum spp. basilicum*, *Melaleuca quinquenervia* CT cineole, *Cinnamomum camphora* CT cineole, and *Rosmarinus officinalis* CT cineole) were furnished by Pranarôm Science, France. Major components of these EOs are listed in Table 1.

### 2.2. Bacterial Strains and Culture Conditions

*Streptococcus pyogenes *CIP 104226 strain was used in this study (Collection de l'Institut Pasteur, France). The strain was clinically isolated from pharynx of a child following episode of pharyngitis. 

### 2.3. Disk Diffusion Assay

Antimicrobial activity was investigated by disc diffusion method as already described [5]. The bacterial suspension was adjusted to a density of bacterial cells of 1.0 × 10^8^ UFC/mL (or 0.5 McFarland turbidity units). A sterile swab immersed in this bacterial suspension was used to inoculate the entire surface of a sheep blood agar (Biomerieux). 6 *μ*L of each EO was applied on a sterile paper disc (Biomerieux) aseptically placed on the inoculated plates. Then, plates were incubated for 15 minutes at room temperature. Only one disc was tested per plate. After 24 h of incubation at 37°C in a CO_2_ incubator, the inhibition zones were measured in millimetres. Amoxicillin (25 *μ*g/disc, Bio-Rad) was used as a positive control for bacterial inhibition. All experiments were done in triplicate. The average of inhibition diameters was calculated to classify the EOs as follows: *S. pyogenes* is not sensitive (0) for a diameter smaller than 8 mm, moderately sensitive (+) for a 8–14 mm diameter, sensitive (++) for a 14–20 mm diameter, and very sensitive (+++) for a diameter larger than 20 mm [5, 6].

### 2.4. Determination of Minimum Inhibitory Concentration and Minimum Bactericidal Concentration

Essential oils with a large inhibition diameter (>20 mm) were examined for their antimicrobial activity against *S. pyogenes. *The Minimum Inhibitory Concentration (MIC) was estimated by the broth dilution method in Brain-Heart broth (BH, Biomerieux) using the standardized method described by Courvallin et al. [7]. Briefly, each EO was first diluted in DMSO (diméthylsulfoxyde): 40% (v/v) for *Cinnamomum verum* and 80% (v/v) for the other EOs tested. Serial dilutions of EOs were carried out in distilled water with concentrations ranging from 0.025% to 1% (v/v), depending on the EO tested. One milliliter of a *S. pyogenes *inoculum (10^6^ UFC/mL) and 0.1 mL of each EO dilution were added to 2.9 mL of Brain-Heart broth. Controls without EO were prepared. After 24 h of incubation at 37°C under agitation, on hermetic tubes, MIC was determined as the lowest concentration of the EO inhibiting visible bacterial growth. 

To determine the Minimum Bactericidal Concentration (MBC), 10 *μ*L of bacterial inoculum was taken aseptically from tubes that had not presented visible turbidity and was plated onto sheep blood agar [7]. The MBC was considered as the lower concentration of EOs that allowed less than 0.1% of the original inoculum treated with the EO to grow on the surface of the sheep blood agar. Each MIC and MBC value was obtained from three independent experiments. To determine the nature of antibacterial effect of EOs, the MBC/MIC ratio was used; when the ratio was lower than 4, the EO was considered as a bactericidal EO and when the ratio was higher than 4, it was considered as a bacteriostatic EO [8].

## 3. Results

### 3.1. Essential Oils Composition

As depicted in Table 1, essential oils were chosen according to their chemical composition, in particular to their major components. Major compounds of *Cinnamomum verum* and *Cymbopogon citratus* were aldehydes. *Origanum compactum*, *Thymus vulgaris *CT thymol, *Satureja montana*, *Eugenia caryophyllus*, and *Ocimum basilicum *mainly contained phenolic derivatives. Analysis of *Cymbopogon martinii var. motia*, *Cinnamomum camphora *CT linalool, *Mentha piperita*, *Thymus vulgaris *CT thujanol, *Origanum majorana*, and *Melaleuca alternifolia* indicated terpene alcohols. Ketones were major compounds from *Lavandula stoechas*. At least, as indicated by their chemotypes, *Melaleuca cajuputi*, *Melaleuca quinquenervia* CT cineole, *Cinnamomum camphora *CT cineole, and *Rosmarinus officinalis *CT cineole mainly contained cineole, a monoterpene ether.

### 3.2. Screening of Antibacterial Activity

**Figure 1:**
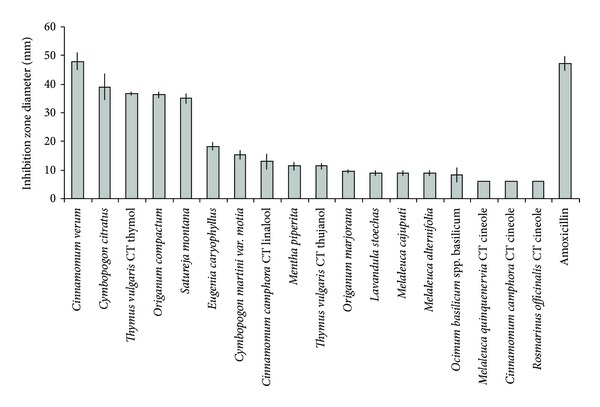
Inhibition zone diameters obtained with the various essential oils against *S. pyogenes *(means ± SD).

Results obtained with disk diffusion assay regarding the growth inhibition zones of the tested *S. pyogenes* strain are presented in Figure 1. Our results showed that EOs from *Cinnamomum verum, Cymbopogon citratus, Thymus vulgaris *CT thymol*, Origanum compactum*, and* Satureja montana *are the most active oils tested against *S. pyogenes*, with inhibition zones average ranging from 48.0 mm to 35.0 mm (+++). *S. pyogenes* is sensitive (++) to *Eugenia caryophyllus* and *Cymbopogon martinii var. motia* (means inhibition diameters, resp., 18.3 and 15.3 mm). Most of EOs tested showed a moderate inhibitory activity (+) against *S. pyogenes* (means inhibition diameters ranging from 13.0 to 9.0 mm): *Cinnamomum camphora *CT linalool*, Mentha piperita, Thymus vulgaris *CT thujanol*, Origanum majorana, Lavandula stoechas*, *Melaleuca cajuputi, Melaleuca alternifolia. *Four EOs showed no significant activity (0) against the tested strain (inhibition zone diameter ranging from 6.3 to 0.0 mm): *Ocimum basilicum spp. basilicum*, and* Melaleuca quinquenervia *CT cineole*, Cinnamomum camphora *CT cineole, and *Rosmarinus officinalis *CT cineole. Inhibition zones of almost all the essential oils were significantly lower than the positive control amoxicillin (47.3 ± 2.5 mm). 

### 3.3. MIC and MBC Values Determination

**Figure 2:**
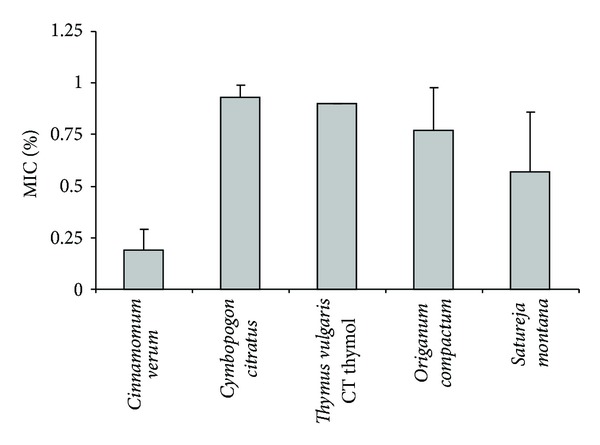
Minimum Inhibitory Concentrations of five selected essential oils against *S. pyogenes* (means ± SD).

Referring to the large inhibition zones observed with disk diffusion method for five essential oils (*Cinnamomum verum, Cymbopogon citratus, Thymus vulgaris *CT thymol*, Origanum compactum*, and* Satureja montana*), the MIC values were determined with broth dilution assays (Figure 2). *Cinnamomum verum *EO mainly composed of aromatic aldehyde was the most efficient against *S. pyogenes* (0.19% (v/v)). MIC of *Cymbopogon citratus* containing mainly terpenic aldehyde was 0.93% (v/v). As far as the EOs rich in aromatic phenols were concerned, MICs ranged from 0.57 to 0.90% (v/v). 

Concerning MBC, in most cases, it was close to the MIC, indicating a good bactericidal activity against *S. pyogenes *(Table 2), with a ratio of MBC to MIC ranging from 1.02 to 1.53.

## 4. Discussion 

Even if aromatic and medicinal plants have been used from ancient times as natural therapies and are considered as alternatives to synthetic drugs, scientific investigations to evaluate antimicrobial activity of essential oils are needed.

The aim of this work was to focalize in EOs usable against *S. pyogenes* and to compare their antimicrobial activity specifically against *S. pyogenes*, a bacteria responsible for human tonsillitis. Eighteen EOs have been selected for their composition. Indeed, in the literature, it has been reported that EOs containing mainly aromatic phenols or aldehydes presented a major antimicrobial activity against respiratory tract pathogens, followed by EOs containing terpene alcohols. EOs containing terpene ether, ketone, or oxide had weaker activity [9, 10]. Then, for example, thyme, cinnamon, lemongrass, tea tree, lavender, oregano, clove, palmarosa, or cajeput EOs are known to be active against *S. pyogenes* [9–12] while oregano, basil, mint, rosemary, and lavender EOs are known to inhibit another Gram-positive bacteria, *Staphylococcus aureus *[13]. 

In this study, we used the standardized disk assay method to select 5 essential oils showing the higher inhibitory activity against *S. pyogenes* among 18 essential oils tested. The obtained results, in accordance with the literary works, showed that EOs mainly composed of aldehydes or phenols were the most effective against *S. pyogenes.*

**Figure 3:**
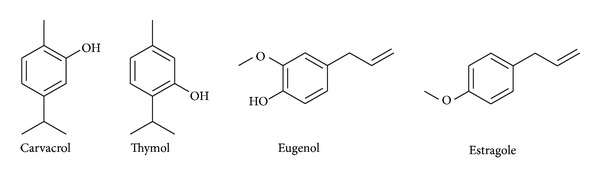
Structure of phenolic derivatives from tested essential oils.

We showed that cinnamon presented the higher activity against *S. pyogenes* compared to the other EOs tested. These results were consistent with previous works [10, 11]. Therefore, *Cinnamomum verum* EO containing cinnamaldehyde (an aromatic aldehyde) showed the highest activity. Moreover, EOs containing the aromatic phenols, carvacrol and thymol, were very efficient (+++) against the *S. pyogenes* strain tested. The other oils containing a phenolic derivative (clove containing eugenol and basil containing estragole) were less active (++). As depicted in Figure 3, these results could be directly linked with the structures of major aromatic phenolic derivatives from EOs. Particularly, the presence of free phenol seems to increase antibacterial activity against *S. pyogenes*. Basil EO contains mainly estragol without any free phenol. It can be surprising that clove was not selected among the essential oils showing the stronger antibacterial activity. Indeed, previous studies had shown that antibacterial activity of clove EO against *S. pyogenes* was nearly the same as thyme EO [11]. The differences between our results and previous studies can be due to the fact that the composition of EOs is not strictly defined but is a complex mixture of organic substances, varying in quality and quantity [14].

Antibacterial activity of the 5 oils selected was studied by determining the MIC and the MBC. In this study, results of MICs were reliable with diameters of inhibition zones observed with disk diffusion method, with *Cinnamomum verum *being the more effective EO followed by the other four EOs tested. All tested EOs showed bactericidal activity *in vitro* (MBCs nearly equal to MICs) but investigations such as pharmacokinetic and pharmacodynamic studies are needed to characterize the antibacterial activity *in vivo* and their clinical efficacy [15]. 

The comparison of antibacterial activity and cytotoxicity of EOs and antibiotics must be approached with caution. Indeed, due to the complex and variable chemical composition of EOs, affected by many factors like chemotypes or cultivation conditions, it is still difficult to understand antibacterial activity mechanisms of EOs and to control their cytotoxicity. As discussed later, at least one part of the antibacterial and cytotoxic activities of essential oils is nonspecific but linked with lipophilic compounds targeting membranes.

Screening the EOs presenting antibacterial effects, Fabio et al. have reported that MICs of the EOs showing antibacterial activity were higher than the nontoxic concentrations on Vero cells [11]. Investigations on cytotoxicity of EOs have to be conducted, particularly in terms of possibility of overdoses and in terms of interactions with drugs. Moreover, the lack of clinical studies (toxicity, pharmacokinetic, etc.) has to be underlined. 

Therapeutic doses of EOs containing phenols or aldehydes are usually only a few drops per day (2 drops 3 times a day) *per os*. The inhalation of such irritating EOs should be avoided. It should be noticed that pharmacokinetic predictions about mixtures such as EOs are difficult [14]. Nevertheless, as essential oils are lipophilic and volatile compounds, they can rapidly reach the systemic compartment and be partially eliminated through respiratory way, that is, on the infectious site in tonsillitis context [16].

Antibacterial treatment of *S. pyogenes* tonsillitis has several objectives; among them is the reduction of the transmission to family members. Indeed, it seems that there is an increased risk concerning the invasive infections (e.g., bacteraemia and pneumonia) for household contacts of index patients, compared to the annual incidence rate of sporadic invasive infections caused by this bacteria [17]. However, about 20–30% of antibiotic treatments with penicillin fail to eradicate the pathogen [3, 4]. This failure in therapy is not due to a resistant phenotype to penicillin [18] but can be related to various hypothesis such as microbiologic interactions between commensal pharyngotonsillar flora and *S. pyogenes*, poor penetration and diffusion of penicillin into tonsils, or reacquisition of *S. pyogenes* from a contact [2]. In this context, synergy of action of EOs with antibiotics could be investigated. With a nonspecific mode of action, EOs could help to control beta-lactamase-producing bacteria causing failure of penicillin treatment to eradicate *S. pyogenes* (for a review see [19]). 

An illustration of this promising strategy of combination is the study performed by Fadli et al. Among 80 EOs/antibiotics combinations tested, 71% showed synergism. For example, it has been noted that carvacrol showed a synergistic effect when combined with ciprofloxacin [20]. Moreover, this alternative strategy could be interesting because it can ideally lead to a reduction of doses of the antibiotics, thus reducing the adverse effects of the therapy. However, the synergistic effects have to be evaluated *in vitro* and *in vivo* as effects are variable, depending on many factors like EO composition, exposure time, or mode of action of the active components of the EO. Investigations on EOs mechanisms of action have not been fully established yet. Studies on *Cinnamomum *sp. and on its major components showed morphological changes (loss of membrane integrity) on Gram-positive bacteria (*Staphylococcus aureus *or *Bacillus cereus)* and a decrease in the metabolic activity and in the bacterial replication capacity [21, 22]. Carvacrol exposure causes morphological modifications, as changes in cell surface structure [23, 24]. However, as EOs components are lipophilic, membranes of various organisms are likely targeted but protein targets seem nonspecific; particularly, phenolic alcohols or aldehydes interfere with membrane-integrated or -associated enzyme proteins, stopping their activity. Components of EOs can also interfere with electron transport chain from bacterial or mammalian mitochondria and alter energy production [25].

## 5. Conclusion

In conclusion, we show an interesting antibacterial activity of some essential oils against *S. pyogenes*, particularly *Cinnamomum verum* EO but we need further investigations to evaluate bactericidal properties in practical applications on clinical strains and to assess the potential for therapeutic application. Fronting the fact that there is no evidence of a potential clinical use of these EOs, further researches are needed in order to determine if they could substitute efficiently antibiotics or, perhaps, be used in combination. Additional *in vivo* studies and clinical trials would help to justify and evaluate the potential of these oils in tonsillitis context.

## Figures and Tables

**Table:** (a)

	Major components classified by organic functions	Botanical name—part of the plant (lot number)								
		*Cinnamomum verum*—bark (CVB12)	*Cymbopogon citratus*—aerial part (CCH11)	*Origanum compactum*—flowering top (OCH11)	*Thymus vulgaris* CT thymol—flowering top (TV6H9)	*Satureja montana*—flowering top (SMH11)	*Eugenia caryophyllus*—bud (ECF9)	*Cymbopogon martinii *var.* motia*—aerial part (OF0527)	*Cinnamomum camphora* CT linalool—wood (HOB9)	*Mentha piperita*—leaves (MPH29)
Aldehydes	E-cinnamaldehyde	**65.5**								
	Geranial		**43.4**							
	Neral		**31.1**							

Phenolics derivatives	Carvacrol			**41.8**	2.7	**50.0**				
	Estragole									
	Eugenol	6.2					**81.4**			
	Thymol			16.2	**43.6**	6.8				

Terpene alcohols	Borneol									
	Geraniol		4.3					**82.0**		
	Linalool	3.2			4.9			3.2	**98.5**	
	Menthol									**43.4**
	Myrcenol									
	Neomenthol									4.3
	*α*-terpineol									
	Terpinen-4-ol									
	Cis-thujanol									
	Trans-thujanol									
	Viridiflorol									

Ketones	Camphre									
	Fenchone									
	Isomenthone									3.0
	Menthone									17.6

Terpenes	Camphene									
	*β*-caryophyllene	4.9					6.1			
	p-cymene			11.4	23.5	15.0				
	Limonene		7.1							3.3
	*β*-myrcene					2.5				
	*β*-phellandrene	2.9								
	*α*-pinene									
	*β*-pinene									
	Sabinene									
	*α*-terpinene									
	*γ*-terpinene			16.6	8.2	4.9				
	Terpinolene									
	Bornyl acetate									
	Eugenyl acetate						9.7			

Terpenes	Geranyl acetate		2.4					6.2		
	Linalyl acetate									
	Menthyl acetate									6.2
	Myrcenyl acetate									
	Myrtenyl acetate									

Ethers	1,8-cineole									4.7

**Table:** (b)

	Major components classified by organic functions	Botanical name—part of the plant (lot number)								
		RowSpanEmpty	*Thymus vulgaris*—CT thujanol flowering top (OF0282)	*Origanum majorana*—flowering top (OMH9)	*Lavandula stoechas*—flowering top (OF0625)	*Melaleuca cajuputi*—leave (MCL6)	*Melaleuca alternifolia*—leave (OF0484)	*Ocimum basilicum *spp.* basilicum*—flowering top (OF0761)	*Melaleuca quinquenervia *CT cineole—leave (BMQ1L23)	*Cinnamomum camphora* CT cineole—leave (OF0481)	*Rosmarinus officinalis *CT cineole—flowering top (RO2H14)
Aldehydes	E-cinnamaldehyde									
	Geranial									
	Neral									

Phenolics derivatives	Carvacrol									
	Estragole						**70.9**			
	Eugenol									
	Thymol									

Terpene alcohols	Borneol									3.1
	Geraniol									
	Linalool				3.2		20.6			
	Neomenthol									
	Menthol									
	Myrcenol	9.1								
	*α*-terpineol	2.9	4.5		10.7	2.7		5.6	8.1	
	Terpinen-4-ol	**13.0**	**29.2**			**39.4**				
	Cis-thujanol	5.2	**11.8**							
	Trans-thujanol	**26.0**	2.8							
	Viridiflorol							3.4		

Ketones	Camphre			**31.8**						10.2
	Fenchone			**29.8**						
	Isomenthone									
	Menthone									

Terpenes	Camphene			4.4						4.3
	*β*-caryophyllene									3.3
	p-cymene					3.0				
	Limonene	3.0			5.3			7.8		2.2
	*β*-myrcene	5.1								
	*β*-phellandrene									
	*α*-pinene	2.9		3.6		2.6		9.3	5.0	10.3
	*β*-pinene							2.4	3.5	8.5
	Sabinene	2.3	6.4						14.9	
	*α*-terpinene	3.9	7.2			9.2				
	*γ*-terpinene	6.6	12.6			20.8				
	Terpinolene		2.8			3.3				
	Bornyl acetate			3.8						
	Eugenyl acetate			4.0						
	Geranyl acetate									
	Linalyl acetate		2.2							
	Geranyl acetate									
	Menthyl acetate									
	Myrcenyl acetate	3.4								
	Myrtenyl acetate			3.7						

Ethers	1,8-cineole				**60.3**	2.8		**58.7**	**53.8**	**44.5**

**Table 2:** Minimum Bactericidal Concentrations and MBC/MIC ratio of five selected essential oils against *S. pyogenes*.

	MBC*	MBC/MIC
*Cinnamomum verum *	0.25 ± 0	1.32
*Cymbopogon citratus *	0.95 ± 0.07	1.02
*Thymus vulgaris *CT thymol	0.87 ± 0.15	1.13
*Origanum compactum *	0.97 ± 0.06	1.08
*Satureja montana *	0.87 ± 0.15	1.53

*Means of MBCs ± SD.
